# Identifying Early Indicators of Tail Biting in Pigs by Variable Selection Using Partial Least Squares Regression

**DOI:** 10.3390/ani13010056

**Published:** 2022-12-23

**Authors:** Veronika Drexl, Imme Dittrich, Thore Wilder, Sophie Diers, Joachim Krieter

**Affiliations:** 1 Institute of Animal Breeding and Husbandry, Christian-Albrechts-University, Olshausenstraße 40, D-24098 Kiel, Germany; 2 Chamber of Agriculture of Schleswig-Holstein, Gutshof 1, D-24327 Blekendorf, Germany

**Keywords:** rearing, fattening, partial least squares regression, tail biting, early intervention

## Abstract

**Simple Summary:**

Tail biting is one of the major animal welfare issues in pig farming and can be avoided or reduced by early detection using various indicators. The aim of the study was to identify the most important variables from a set of pen-level (tail posture, daily health control, treatment index, weight, water consumption and activity time) and environmental variables (temperature, humidity, NH_3_ and CO_2_ concentration, exhaust air rate and outdoor temperature). In rearing and fattening, variables were collected either by direct observation or by using sensors. Variables were selected mainly from the pen-level variables in rearing. In fattening, environmental and pen-level variables were selected. This indicates that in rearing the environmental aspects have less influence on the development of tail lesions than in fattening. Nevertheless, the most relevant variable in both rearing and fattening is tail posture. The selected variables have contributed to the explanation of the variance of tail lesions; thus, these can be used as predictor variables for the early detection of tail biting in further investigations.

**Abstract:**

This study examined relevant variables for predicting the prevalence of pigs with a tail lesion in rearing (REA) and fattening (FAT). Tail lesions were recorded at two scoring days a week in six pens in both REA (10 batches, 840 scoring days) and FAT (5 batches, 624 scoring days). To select the variables that best explain the variation within the prevalence of pigs with a tail lesion, partial least squares regression models were used with the variable importance in projection (VIP) and regression coefficients (β) as selection criteria. In REA, five factors were extracted explaining 60.6% of the dependent variable’s variance, whereas in FAT five extracted factors explained 62.4% of the dependent variable’s variance. According to VIP and β, seven variables were selected in REA and six in FAT with the tail posture being the most important variable. In addition, skin lesions, treatment index in the suckling phase, water consumption (mean), activity time (mean; CV) and exhaust air rate (CV) were selected in REA. In FAT, additional musculoskeletal system issues, activity time (mean; CV) and exhaust air rate (mean; CV) were selected according to VIP and β. The selected variables indicate which variables should be collected in the stable to e.g., predict tail biting.

## 1. Introduction

Tail biting is one of the most important issues in pig welfare the causes of which are multifactorial [1,2]. When pigs cannot meet their biological needs or are stressed by nutritional, environmental, management or health issues, tail biting can be triggered [3,4]. Risk factors for tail biting are distinguishable into constant factors, which are given by the husbandry conditions, and changeable factors, which change in response to the environment. Constant risk factors are for instance space availability [5], the feeding system [6], the availability of occupation material [2] as well as genetics [7]. Changeable risk factors within the rearing (REA) or fattening (FAT) phases are indicators concerning the health of the pigs [7,8] or affecting the climatic environment [9,10]. To reduce constant risk factors, the farmer is able to improve the husbandry conditions in the stable. The changeable factors can be observed and analysed to enable direct reactions or rather interventions when alterations occur.

Observable alterations in behavioural patterns for instance indicate stress, which is related to changes in the pigs’ health and the occurrence of tail biting [3,11,12]. Among pen-level indicators as indicators related to pig health, tail posture, disorders of the respiratory tract, the musculoskeletal system as well as weight and daily weight gain were determined [5,13,14,15]. Furthermore, behavioural changes in pigs are measurable in changes of water consumption as well as pig activity and are linked to the development of tail biting [12,16,17,18].

Besides the indicators mentioned, changes in the environmental conditions, e.g., unsteady climates with large temperature changes and exhaust air rate with draughts, are also considered to be risk factors of tail biting [9,19,20]. The impact of indoor and outdoor temperature on the development of tail lesions was suggested [1,2], which was proved for NH_3_ concentrations [21,22,23]. On the other hand, temperature, humidity and exhaust air rate had an influence on activity [24], which is a known influence on the development of tail lesions [17,18].

Several relationships between tail lesions and factors contributing to tail lesions have been identified and other associations, e.g., pig health, have been indirectly verified [3]. The presence of numerous variables as potential indicators causes a demand for the identification of the variables that contribute to the development of tail lesions from a set of pen-level and environmental variables. A precision livestock system for the early detection of tail biting could be developed and used as early-warning system considering the indicators mentioned [25]. Thus, the aim of the present study was to select variables affecting tail lesions. To assess the practicability of the application in stables, the prevalence of pigs with a tail lesion in rearing and fattening were determined at pen level and the variables influencing them selected. The selection was carried out using partial least squares regression models using the selection criteria variable importance in projection and regression coefficients at defined limits.

## 2. Materials and Methods

### 2.1. Animals and Housing

Data were collected from February 2020 to October 2021 at the research farm of the Chamber of Agriculture of Schleswig-Holstein in Futterkamp, Germany. Tail biting behaviour was investigated in undocked and uncastrated crossbred pigs (Pietrain × (Large White × Landrace)) from the day of weaning to slaughter. Overall, 1280 pigs were reared in ten batches and 613 pigs were fattened in five batches. The pigs were kept in two differently designed compartments, one compartment being barren and one being enriched in both REA and FAT. While the barren compartments included two pens with room for 28 pigs each, the enriched compartments contained of four pens with room for 18 pigs each. As the pigs were separated by sex, in both barren compartments, one pen was provided for a group of male and the other pen was provided for a group of female pigs. The enriched compartment had two pens with male pigs and two pens with female pigs. The pigs were weaned after an average suckling phase of 28 days (average weaning weight: 8.6 kg ± 0.92 kg) and transferred to FAT after 47 days of REA (average weight: 32.0 kg ± 4.41 kg). The pigs were slaughtered after 166.4 days ± 7.67 days on average with an average live weight of 116.7 kg ± 7.56 kg. After rearing, the pigs were transferred into FAT without mixing the groups; hence, the pigs were fattened in their rearing groups. Consequently, the pigs were familiar with their pen mates and did not have to deal with e.g., rank fights. The housing conditions were in accordance with the EU Council Directive 2008/120/EC, EU Council Directive 2010/63/EC and the ‘German Order for the Protection of Production Animals used for Farming Purposes and other Animals kept for the Production of Animal Products’ (TierSchNutztV, 2017). As approved by the ethics committee in charge, an ethical approval of the protocol was not mandatory for the present study.

**Figure 1:**
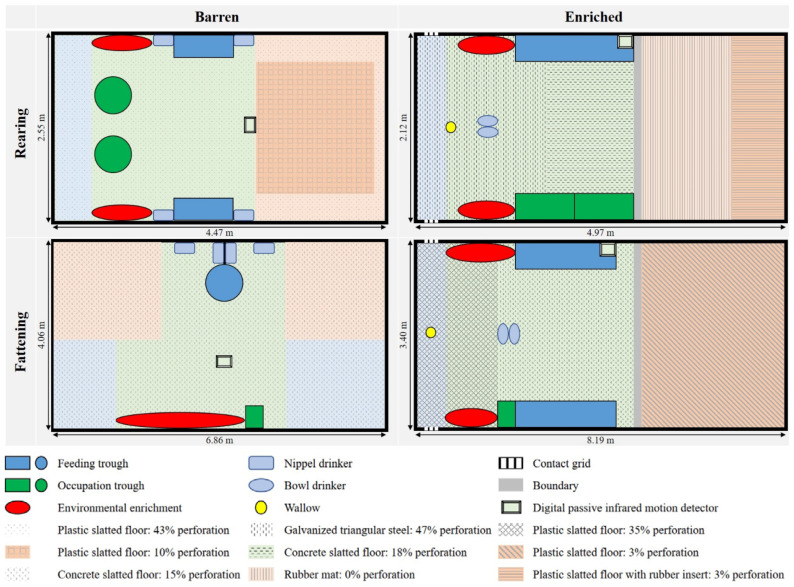
Illustration of the barren and enriched pens in rearing and fattening. The colours indicate the pen structure: blue for the faeces area, green for the activity area and orange for the lying area.

In REA, the space allowance per pig was 0.4 m^2^ in the barren and 0.5 m^2^ in the enriched pens. Plastic slatted floors were installed in the barren pens in REA (Figure 1), with a perforation of 43% in the faeces and activity area as well as around the lying area; the floor in the lying area had a perforation of 10%. Water was provided by four nipple drinkers in each pen. While two short troughs were available for rearing feed mixture (ad libitum milled dry feed), two additional round troughs were installed for the occupational feed. The floors in the enriched pens in REA (Figure 1) were galvanised triangular steel (47% perforation) in the faeces area, a concrete slatted floor (18% perforation) in the activity area and a rubber mat (0% perforation) as well as a plastic slatted floor with a rubber insert (3% perforation) in the covered lying area. The lying area was separated from the activity area by a threshold. Two bowl drinkers provided water. A long trough was available for rearing feed mixture (ad libitum granulated dry feed) and two short troughs were additionally installed for occupational feed. Furthermore, a wallow and a contact grid were available. In REA, the animal to feeding place ratio was 3:1 in the barren pens and 1:1 in the enriched pens.

In FAT, 1.0 m^2^ was available for each pig in the barren pens and 1.5 m^2^ was available in the enriched pens. The barren pens in FAT (Figure 1) were provided with concrete slatted floors (15% perforation) and four nipple drinkers as well as a mash feeder for the fattening feed mixture and a dry feeder for the occupational feed. In the enriched pens in FAT (Figure 1), plastic slatted floor was installed in both the faeces and the covered lying area with a perforation of 35% and 3%, respectively. Again, a threshold was installed to separate the lying area from the activity area that was equipped with a concrete slatted floor (15% perforation). In these pens, water was provided by two bowl drinkers and the fattening feed mixture (dry feed) was provided ad libitum in two long troughs as well as a dry feeder for occupational feed. Again, a wallow and a contact grid were available in the enriched pens. In FAT, the animal to feeding place ratio was 7:1 in the barren pens and 2:1 in the enriched pens.

As occupation material, cotton ropes (Ø 8 mm) and coniferous wood (8 cm × 8 cm) were available in a hanging position (Figure 1) in the pens at all times. In addition, in each pen, occupational feed was supplied ad libitum and was composed as a mixture of grain maize, field beans, peas, spelt husk pellets (in REA) or grass pellets (in FAT). The occupational feed was replenished twice a day at the same time as the provision of chopped straw in the lying area of the enriched pens of REA and FAT. As an intervention action in cases of tail biting (fresh tail lesions in comparison to the day before), jute sacks were provided as additional occupation material. If biters or victims were identified, these were removed as a further intervention measure. Overall, 21 biters and 2 victims were removed within REA as well as 4 biters within FAT.

The temperature within the REA period was continuously adapted to the pigs’ needs and requirements. Hence, in the barren pens, the temperature was set to 29 °C at the beginning of REA and then successively reduced to 22.7 °C at day 40 of REA. In the enriched pens, the temperature was set to 23 °C at the first day of REA and successively lowered to 19 °C at the end of REA. Under the cover of the lying area, the temperature started with 29 °C at the beginning and ended with 23 °C at the end of REA. In FAT, the temperature in the barren pens started at 23 °C and changed successively to 17.8 °C at day 70 of FAT. In the enriched pens of FAT, the temperature was set 18 °C at the beginning that was gradually decreased to 17 °C at day 21 until the end of FAT. The lights were automatically switched on at 6 am and switched of at 6 pm with 170 lux (barren pens) and 350 lux (enriched pens), respectively, in REA and 320 lux (the barren pens) and 240 lux (enriched pens), respectively, in FAT.

### 2.2. Data Collection

With regard to tail lesions, in REA and FAT, the pigs were scored twice a week in the morning, whereby the time interval between two scoring days was named period t. Hence, in each batch 14 scoring days were recorded in REA and 24 scoring days were recorded in FAT until the first pigs were taken to the slaughterhouse. In the first batch of FAT, severe tail biting was registered, and the pigs were regrouped and rehoused due to ethical reasons; thus, data collection stopped after 4 weeks and 8 scoring days in this particular batch. From REA, 840 scoring days (10 batches, 14 scoring days each, 6 pens) and from FAT 624 scoring days (4 batches, 24 scoring days each, 6 pens and 1 batch, 8 scoring days, 6 pens) were available for analysis.

In accordance with the type of collection, data are differentiable into direct observations and sensor data variables that were recorded at pen level except for the environmental variables, which were partially recorded at compartment level (e.g., NH_3_ concentration) and used for all pens of the compartment.

The direct observations variables are described in Table 1. On the scoring days, both tail lesions and tail posture of the pigs were collected. First, the tail posture was scored from outside the pen followed by entering the pen for the scoring of the tail lesions. The tail posture was recorded in five categories using a modified scheme of Kleinbeck and McGlone [26] and summarised into binomial data as follows: “0” indicates a lifted tail posture of curled and raised tails and “1” indicates a lowered tail posture of wagging, hanging and jammed between the hind legs. The four categories of tail lesions were scored according to the ‘German Pig Scoring Key’, German designation: Deutscher Schweine-Boniturschlüssel [27]. The tail lesions were summarised into a binomial scheme: “0” represents the category “no lesion”, which includes all intact tails and tails with superficial lesions; “1” refers to the category “lesions” and includes all tails with small and large lesions. The scoring described was carried out by an independent observer. Daily animal controls were carried out by staff throughout the week (Monday to Friday) in the morning, determining the proportion of pigs per pen showing skin lesions or respiratory (cough), gastro-intestinal (diarrhoea) and musculoskeletal (lameness) issues. Pigs with respiratory issues were counted from outside the pen, these numbers were used to calculate the proportion of pigs with respiratory issues for each pen. Subsequently, the pen was entered to record the proportion of pigs with other issues. The frequency tail lesions, tail posture and the observations of the daily control variables from each pen were related to the number of animals to determine the prevalence per pen. A treatment index was determined for the suckling phase, REA and FAT, in which all treatments were included except for the ones due to tail biting. The pigs were weighed at the day of weaning and at the end of REA.

Sensor data variables are explained in Table 2. In each pen, water consumption was measured hourly. A digital passive infrared motion detector was installed in each pen (Figure 1) to record the activity of the pigs. The sensor was used at pen level and detected moving pigs and still pigs, which were labelled as a ‘true’ or a ‘false’. The data were recorded as “1” for activity in the pen and “0” for no activity in the pen. The activity as well as a temperature and humidity sensor, which was installed in each pen next to the drinker, stored the data whenever changes within activity, temperature or humidity occurred. These sensor data were processed to provide a value for each minute of the day. The temperature and humidity were used to determine the temperature humidity index. NH_3_ and CO_2_ concentrations were recorded every 15 min (96 measurements a day) in the middle of one pen per compartment. The ventilation computers of the compartments recorded exhaust air rate as a measure of air removed from the room and outside temperature every minute. The above mentioned set temperature was achieved by an automatically controlled regulation of the exhaust air rate and the heating depending on the outdoor temperature particularly at the beginning of REA and FAT.

All variables between two scoring days, in period t, were aggregated and allocated to the following scoring day. Scoring day 1 in rearing and fattening was omitted, as no prior information was available. For example, if in a week the scoring days were conducted on Monday and Thursday morning, the data from Monday to Wednesday were aggregated and allocated to the Thursday scoring day. Similarly, the data from Thursday to Sunday were aggregated and allocated to the scoring day on the following Monday, except for the tail posture data, for which the tail posture of the scoring day was assigned to the tail lesions of the following scoring day.

### 2.3. Statistical Analysis

A variable selection was chosen to determine the variables contributing to the prediction of tail lesions. A partial least squares (PLS) regression model was used to assess which set of n variables X_(n)_ could predict the dependent variable y_(n)_. Based on Mehmood et al. [28], a linear relationship y_(n)_ = α + X_(n)_ β + ε is presumed between the predictors (X) and the dependent variable (Y), whereas the regression parameters α, β and the error term ε are unknown. The dependent and independent variables are previously centred and scaled to mean = 0 and standard deviation = 1 [28]. The prediction of one or more dependent variables is possible with a PLS regression model even with a large number of predictors and a low number of observations [29]. Thus, the PLS regression models were calculated using the PLS procedure of the statistical analysis software SAS^®^ 9.4 [30] with the option ‘leave-one-out cross validation’.

The information of all measured variables of the (3–4) days before a scoring day were used to estimate the prevalence of pigs with a tail lesion at pen level. The prevalence of pigs with a tail lesion was 11% for REA (21% in the barren pens, 7% in the enriched pens) and 16% for FAT (in both pen types). For REA and FAT, the dependent variable was the prevalence of pigs with a tail lesion considering both pen types together and the independent variables were the variables described in Table 1 and Table 2, with the scoring day being added as a class variable.

**Table 2:** Pen-level and environmental variables were direct observations or sensor data. Sensor data variables according to recording time, the value used in the model and the information relating to how the variables were measured or stored. Within t (period between two tail lesions scoring days), the data were aggregated and allocated to the following tail lesions scoring day. Mean and coefficient of variation (CV) were selected as features for the sensor data variables.

Data	Variable	Recoring Time	Value	Measured with	Stored in
Pen level	Water consumption [1]	60 min	Per pig within t	Composite piston meter with pulse module (Lührs Gerätebau GmbH, Rehden, Germany)	Stable PC
	Activity time ^1^		Per pen within t	Aqara Motion Sensor (Item number RTCGQ11LM, Lumi United Technology Co., Ltd., Shenzhen, China)	Single-board computer (Raspberry Pi 3 Model B+ [31]) with a RaspBee add-on board (dresden elektronik, Dresden, Germany)
Environmental	Temperature [°C] ^1^		Per pen within t	Aqara Temperature and Humidity Sensor (Item number WSDCGQ11LM, Lumi United Technology Co., Ltd., Shenzhen, China)	
	Humidity [%] ^1^				
	Temperature humidity index (THI) ^1^			Calculation THI according to Vitt et al. [32] with THI_NOAA_ = 0.81T + 46.3 + H/100 (T−14.3) with T = temperature and H = humidity	
	NH_3_ concentration [ppm]	15 min	Per compartment within t	Polytron C300 with DrägerSensor NH_3_ AL (Dräger, Lübeck, Germany)	ALMEMO 2590-4AS (Ahlborn Mess- und Regelungstechnik GmbH, Holzkirchen, Germany)
	CO_2_ concentration [ppm]			VarioGard 2320 IR CO_2_ PL (Dräger, Lübeck, Germany)	
	Exhaust air rate [%]	1 min		Rearing barren: KL-6002 (Stienen Bedrijfselektronica B.V., Nederweert, Niederlande) Rearing enriched: LC4-C (hdt Anlagenbau GmbH, Diepholz, Germany) Fattening barren: DR1-D (Möller GmbH, Diepholz, Germany) Fattening enriched: DOL 234 (SKOV A/S, Roslev, Denmark)	Stable PC
	Outdoor temperature [°C]				

^1^ The observations of activity, temperature, humidity and temperature humidity index were measured at inconsistent time intervals, as only changes that could occur sporadically were recorded.

Several features were available for the aggregation of the sensor data variables. To determine which features should be used for the sensor data variables in a variable selection model, a feature selection was carried out with regard to the mean values (mean, median and mode) and the scattering parameters (skewness, kurtosis, variance, standard error, range and coefficient of variation). For each sensor data variable, all features were calculated over period t between two scoring days. The features were tested using the above-mentioned PLS regression model and showed that mean and coefficient of variation (CV) were the most appropriate features. The contribution of each variable to the model in terms of the variance explained was indicated by the VIP and the standardised regression coefficients (β) were estimated as well to confirm the selection of the variables. Therefore, as selection criteria for relevant variables, two comparison options were used that are in accordance with Mehmood et al. [28]: a variable importance in projection (VIP) ≥ 1 and a │β│ ≥ β* = median (β)/interquartile range (β) were assumed as selection thresholds.

Scoring days were excluded if missing values over the entire period t between two scoring days occurred in one of the variables; thus, 688 out of 840 observations were used in REA and 480 out of 624 observations in FAT. Due to some missing values in the environmental variables and to assess the impact of the environmental variables, a PLS regression model without environmental variables was calculated for REA and FAT. For this purpose, 762 observations were used in REA and 547 observations were considered in FAT.

## 3. Results

### 3.1. Rearing

**Figure 2:**
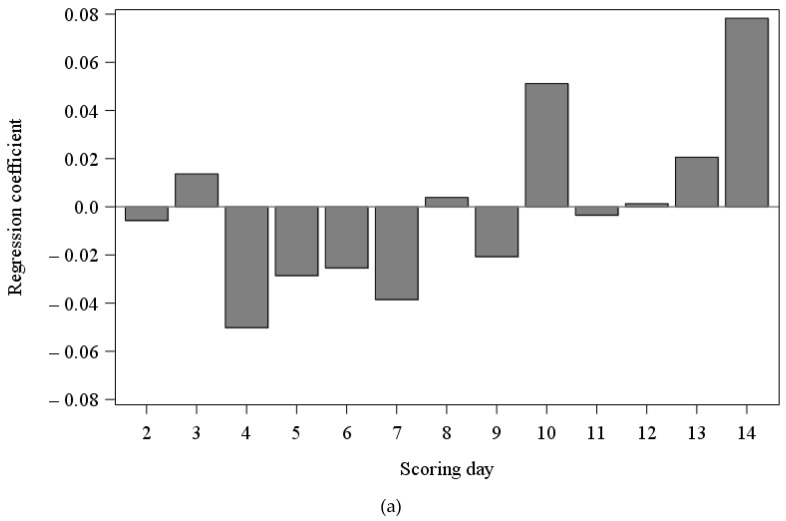

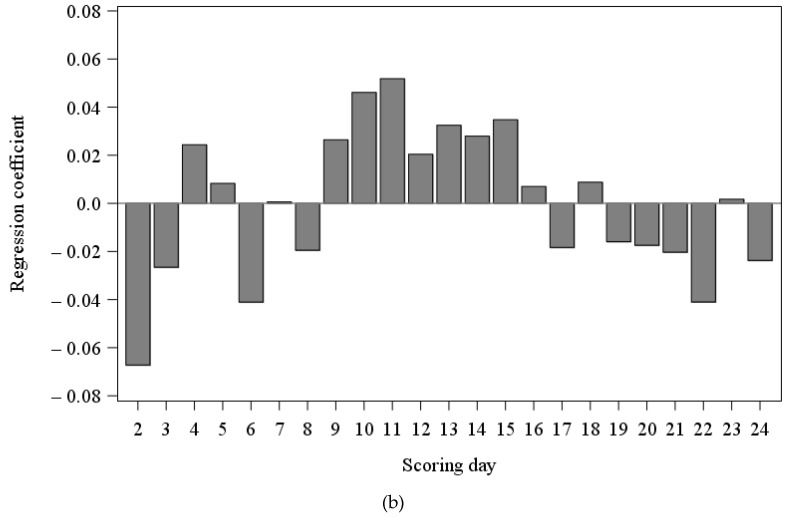
Regression coefficients for the scoring days in rearing (**a**) and fattening (**b**).

All results from the variable selection in REA are presented in Table 3a). In the PLS regression model for REA, five PLS factors were extracted which explained 60.6% of the dependent variable’s variance. For the scoring days within REA, β is shown in Figure 2a). From scoring day 2 until the eleventh scoring day, β showed negative values, except the values changed to positive on scoring day 3 (0.0137), scoring day 8 (0.0039) and scoring day 10 (0.0512). From the twelfth scoring day until the end of REA, β showed positive values with the strongest β on scoring day 14 (0.0783). In REA, ten variables were selected according to VIP and 19 variables according to β. With the strongest β, tail posture (0.6338), skin lesions (0.0736), treatment index in suckling phase (0.1694), water consumption (mean (0.0364)), activity time (mean (0.0584); CV (−0.0379)) and exhaust air rate (CV (−0.0355)) were selected according to both VIP and β. Additionally, treatment index within rearing, water consumption (CV) as well as CO_2_ concentration (mean) were selected according to VIP from the sensor data variables. Respiratory or gastrointestinal tract and musculoskeletal system issues as well as the weight at weaning were selected according to β from the direct observations. Temperature (mean), temperature humidity index (mean; CV), NH_3_ concentration (mean), CO_2_ concentration (CV), exhaust air rate (mean) and outdoor temperature (mean; CV) were selected according to β from the sensor data variables.

The results of the PLS regression model without environmental variables are shown in Table 3a). Four PLS factors were extracted which explained 55.5% of the dependent variable’s variance. The directions and strengths of β (scoring days and variables) are comparable between the two PLS regression models for REA. For the direct observation variables and the sensor data variables from pen level, the same variables were selected according to VIP and β. Only treatment index within rearing was not selected according to VIP; water consumption (CV) was selected according to β.

### 3.2. Fattening

The results from the variable selection in FAT are shown in Table 3b). A proportion of 62.4% of the dependent variable’s variance was explained by five extracted PLS factors in FAT. Figure 2b) presents β for the scoring days within FAT, where β started with negative values on scoring day 2 (−0.0672) and scoring day 3 (−0.0265). Subsequently, β showed mostly positive values until scoring day 15 with several peaks, whereby the strongest values were on scoring day 10 (0.0461) and scoring day 11 (0.0518). A decrease occurred from scoring day 16 until the end of FAT with mostly negative values except on this day, scoring day 18 and scoring day 23. The values decrease until scoring day 22 and continue to increase until the end. In FAT, 15 variables were selected according to VIP and eight variables according to β. According to both VIP and β, tail posture (0.7055) and musculoskeletal system (0.0992) issues were selected from the direct observation variables. Activity time (mean (−0.0826) and CV (0.0690)) and exhaust air rate (mean (−0.0673) and CV (0.0973)) were selected from the sensor data variables according to both VIP and β. In addition, skin lesions, respiratory tract and the weight at the end of rearing were selected from the direct observation variables according to VIP. Water consumption (mean), temperature (CV), temperature humidity index (CV), CO_2_ concentration (mean) and outdoor temperature (mean; CV) were selected from the sensor data variables according to VIP. Humidity (CV) and NH_3_ concentration (mean) were selected additionally according to β.

All results of the PLS regression model without environmental variables are presented in Table 3b). A proportion of 64.7% of the dependent variable’s variance was explained by four PLS factors. The directions and strengths of β between the two PLS regression models for FAT are comparable for the scoring days and the selected variables. Tail posture was selected according to both VIP and β as in the model mentioned previously. The same variables were selected according to VIP except skin lesions, and only tail posture was selected according to β.

## 4. Discussion

### 4.1. Study Design and Statistics

PLS regression models were used to select variables that potentially explain tail lesions in REA and FAT. A selection was required to identify the variables that are most relevant for the model’s outcome, otherwise model performance decreases with increasing complexity [28]. Nevertheless, the collection of a large number of variables is valuable as tail biting is a multifactorial problem and the farmer needs support from state-of-the-art systems in predicting this complex behavioural issue and its consequences, i.e., tail lesions [16]. Furthermore, Larsen et al. [33] found that a single variable is insufficient to reflect a prediction of tail biting. They recommended integrating multiple sets of variables into a predictive model. The aforementioned authors further suggested that a model with constant aspects of the housing conditions is not sufficient for the prediction of tail biting; it is therefore especially necessary to include aspects that show alterations to predict tail biting and tail lesions more precisely. These recommendations are easier to fulfil nowadays as the hardware and software is commonly accessible and available at low costs [34]. However, the most troublesome issue within the use of sensors is represented by missing values, which reduced the number of observations in the present study by 20%. The missing values arose due to sensor failure or the end users during the phase of becoming familiar with the technology [35,36].

The variable selection with PLS regression models is determined by the defined threshold of the selection criteria. In this study, two criteria (VIP and β) were chosen that are based on defined thresholds [28]. Hence, the number of selected variables greatly depends on the defined thresholds of the selection criteria [37]. However, these limits are not necessarily considered to be a strict limitation; thus, variables that are close to the defined thresholds should be selected as well [38]. This particularly applies to the multifactorial character of tail biting [4]; thus, in a generalisation on other farms, more aspects related to the development of tail lesions are represented. A first step in this generalisation is given by the variable selection within the present study, as the results were merely presented without differentiating between the two observed pen types. Nevertheless, the variable selection was carried out for each pen type separately and compared to the more generalised approach. The selected variables from both approaches agreed in most points. Thus, the more generalised approach was chosen. The selected variables were mainly from sensor data variables. The effort required to collect sensor data variables is much lower than that required by direct observation; therefore, more sensor data variables can easily be used.

In the present study, all data collected between the two scoring days were aggregated into single values and assigned to the following scoring day. This procedure is based on the assumption that changes that indicate tail biting are remarkable days before the first tail lesions are observable. However, this approach does not take into account that pigs also modify their behaviour within a day as a reaction to new situations [12,39]. In preparation for the present study, the effect of single day aggregation was tested in comparison to aggregation in period t. For the variable selection investigated in this case, no major differences were observed; thus, aggregation over period t was chosen.

The analysis was carried out at pen level, as the collection of data at pen level is more easily carried out within the daily working routine and less time consuming than individual data collection for pigs [40,41]. However, replacing sensor systems for pen-level observations by systems that are able to observe individual pigs would increase costs due to the number of sensors needed. Consequently, the more practical approach is the collection of group data at pen level [42]. In the future, more animal individual sensors will be applied as observation of individual pigs is possible and promising sensor techniques are currently being investigated [43,44]. To use the data in the development of a PLF system to detect tail biting, the system has to be reliable in prediction of tail lesions. False alarms should be avoided to enhance the farmer’s trust in the system [25,45]. In addition, it is important for the development of a PLF system that it is applicable to other farms and their different husbandry systems [46]. A basis for the development of a general PLF system was the selection of variables in rearing and fattening using barren and enriched pens.

### 4.2. PLS Regression—Variable Selection

#### 4.2.1. Rearing

The values of β over the scoring days in REA are comparable with the course of tail lesions. A negative value at the beginning followed by one positive value at scoring day 3 showed an increase in tail lesions in the second week of REA, which was comparable to Lange et al. [47]. The decrease on scoring day 4, also in the second week of REA, is in accordance with Honeck et al. [48], who observed a comparable decrease in tail lesions in the second week after weaning and ascribed this to rank fighting at the beginning of REA. An increase in β was observed towards the end of REA, which is in accordance to the findings of Veit et al. [49] and Gentz et al. [50], who observed a similar increase in tail lesions in the fourth week of REA, which is comparable to scoring day nine and ten in the present study. The highest values were observed in the current study and by Gentz et al. [50] in the seventh week (scoring day 14) on the last day of REA.

According to the selection criteria, tail posture is the most important variable and achieve the highest VIP values and the strongest β. The strength and direction of the variables’ relation is also indicated by β, which shows a strong positive association between a lowered tail posture and the occurrence of tail lesions. This relationship has been established in other studies [51,52,53]; hence, tail posture is considered to be the most relevant of all variables.

In various studies, impaired pig health was identified as risk factor of tail biting [10,19], and Czycholl et al. [54] identified the impact of the skin status on good pigs’ health. In line with this, skin lesions as a measure of pig health were selected in the present study according to both selection criteria. Another aspect of pig health is the treatment index, whereas the treatment index for the suckling phase was chosen according to both selection criteria and showed high values for both selection criteria. The latter showed a positive β, which indicates that pens with a high treatment index in the suckling phase show more tail lesions. This is potentially related to the circumstance when pigs have experienced disease early in life, they are often unable to compensate afterwards; hence, these pigs tend to be lighter at weaning [55]. This in accordance with other studies that have indicated a tendency for sick as well as lightweight pigs to start tail biting [11,56].

Water consumption and activity were selected according to both selection criteria from the pen-level sensor variables. The use of water consumption for the early detection of tail lesions is in accordance to Domun et al. [16] and Larsen et al. [33], who were able to predict tail biting based on water consumption and environmental such as pen temperature using networks. An association with tail biting and the ability to predict these has also been demonstrated for changes in pig activity [17,18].

In the selection of variables, it is evident that hardly any environmental variables were selected in REA. The PLS regression model without environmental data variables showed that the variance explained by the extracted PLS factors decreased only marginally when excluding these variables. In the sample used in this study, the occurrence of tail lesions and environmental data variables in REA indicates a negligible association. On the one hand, this is in accordance with the literature, since a direct link with environmental data variables in REA has only been proven for NH_3_ [3,21]. On the other hand, in the present study, the ventilation management on the assessed farm was excellent; thus, air quality was likely to have a minor influence on the development of tail lesions.

#### 4.2.2. Fattening

In FAT, the values of β for the scoring days showed negative values at the beginning followed by several peaks in the first weeks of FAT and decreased after scoring day 15 towards the end of FAT, which is comparable to the findings of Gentz et al. [50]. They explained the increase in tail lesions with the beginning of the sexual maturity of uncastrated male pigs, which could have also been an issue in this study. Towards the end of FAT, the pigs became calmer, which is in accordance to Larsen et al. [33], who elaborated decreasing probabilities for tail biting in females and castrated males. These determined the decrease from the seventh week of the FAT, which is comparable to scoring day 12 and 13, whereas in the present study a continuous decline in the values was observed after scoring day 15. This contrast could again have been attributed to the uncastrated male pigs in the current study, as these are generally more aggressive [57,58], and, therefore, more restlessness could have occurred in the pens resulting in more tail lesions.

As tail biting is directly linked to pig health [3,11], the selection of variables for issues with the musculoskeletal system is explicable in the present study according to both selection criteria in FAT. A positive relationship with tail lesions from issues with the musculoskeletal system was observed in the current study as well as in the literature [59,60]. Furthermore, Stygar et al. [61] discovered that treatments of musculoskeletal disorders were related to treatments due to tail biting, supporting the selection of health-related variables in the present study.

Excluding the environmental variables from the PLS regression model explained a comparable amount of the data’s variance as the complete model with all variables. Nevertheless, the environmental variables had a significant impact within FAT with higher risk values [9]. In addition, an influence was identified on a higher prevalence of pigs with a tail lesion of environmental aspects such as the identification of an absence of air turnover in FAT [21]. This indicates the presence of poor air quality and is supported in the present study by the selection of exhaust rate according to both selection criteria. The negative β indicates that fewer tail lesions occurred at high exhaust air rate and, thus, good air quality with the assumption that a better air quality is present when more polluted air is removed by a higher exhaust rate. The exhaust air rate responds to several special variables such as temperature, humidity, NH_3_ or CO_2_ concentration, therefore representing a general variable for air quality.

## 5. Conclusions

This study identified variables in the rearing and fattening of pigs contributing to the explanation of the variance observed in the prevalence of pigs with a tail lesion at pen level. Pen-level and environmental variables were direct observations or sensor data. Variable importance in projection and the regression coefficients were used to select the relevant variables, whereby the most important variable according to both selection criteria in rearing and fattening was tail posture. This was followed in rearing by skin lesions, treatment index in the suckling phase, water consumption and activity time, as well as in fattening with musculoskeletal system issues, activity time and exhaust air rate. To conclude, the selected variables are suitable to explain the variance in the prevalence of pigs with a tail lesion in the rearing and fattening period of pigs.

## Figures and Tables

**Table 1:** Pen-level variables collected by direct observation. Besides the variables, it illustrates the value relating to how the variables were included in the model as well as the classification resp. their definition scheme. The period t is the period between two scoring days; the data between the scoring days were aggregated and allocated to the following scoring day.

Variable	Recording Time	Value	Recorded
Scoring day			Twice a week in the morning over entire rearing and fattening
Tail lesions	Scoring day	Prevalence per pen ^2^	Scoring according to ‘German Pig Scoring Key’ [27]: 0 = No visible lesions or superficial lesions (points or lines of the skin), 1 = Small lesions (deeper skin lesions, smaller than tail diameter) and large lesions (deeper skin lesions, larger than tail diameter)
Tail posture	Scoring day	Prevalence per pen ^2^	Scoring modified after Kleinbeck and McGlone [26]: 0 = Curled (tail forms a loop) and raised (tail lifted, but not curled); 1 = Wagging (tail wagging in motion), and hanging (tail hanging relaxed down) and jammed (tail tucked between the hind legs)
Daily control	Monday–Friday	Prevalence per pen ^2^/mean within t	Proportion of pigs with skin lesions: 0 = No visible skin lesions, 1 = Skin lesions (several, clearly visible scratches)
			Proportion of pigs with respiratory tract issues: 0 = No cough detectable, 1 = Cough (audible or visual)
			Proportion of pigs with gastrointestinal tract issues: 0 = No visible diarrhoea/dirty anal area, 1 = Diarrhoea/dirty anal area (liquid faeces)
			Proportion of pigs with musculoskeletal system issues: 0 = No lameness visible, 1 = Lameness (altered gait)
Treatment index ^1^	Suckling		Entire suckling phase
	Rearing		All treatments within rearing until the respective scoring day in rearing model, entire rearing phase in fattening model
	Fattening		All treatments within fattening until the respective scoring day
Weight [kg]	Weaning	Mean per pen	On weaning day after 28 days of suckling
	End of rearing		On the last day of rearing after 47 days of rearing

^1^ Treatment index = sum treatment units per pen/number of pigs per pen, with treatment units = number of pigs treated × number of treatment days × number of active ingredients (according to QS Qualität und Sicherheit GmbH, Bonn, Germany); all treatments except for treatments due to tail biting. ^2^ Calculation of prevalence per pen: each animal was checked for the presence of 0/1; then, the sum per pen was computed and divided by the number of animals.

**Table 3:** Variable importance in projection (VIP) and regression coefficient (β) for pen-level and environmental variables collected by direct observation or by using sensors for rearing (**a**) and fattening (**b**) and calculated for all variables and without environmental variables. The sensor data variables were tested as mean and coefficient of variation (CV).

(a)						
			**All Variables**		**without Environmental** **Variables**	
			**R^2^ = 0.61**		**R^2^ = 0.55**	
			**β = 0.0123 ^1^**		**β = 0.0032 ^1^**	
**Recorded**	**Variable**	**Feature**	**VIP**	**β**	**VIP**	**β**
Direct observations— Pen	Tail posture		**3.698**	**0.6338**	**3.190**	**0.6436**
	Skin lesions		**1.385**	**0.0736**	**1.258**	**0.0864**
	Respiratory tract		0.382	**−0.0308**	0.363	**−0.0509**
	Gastrointestinal tract		0.469	**−0.0667**	0.436	**−0.0764**
	Musculoskeletal system		0.374	**0.0362**	0.263	**0.0223**
	Treatment index suckling		**1.655**	**0.1694**	**1.143**	**0.1291**
	Treatment index rearing		**1.033**	−0.0013	0.825	0.0005
	Weight weaning		0.827	**0.0511**	0.741	**0.0327**
Sensor data—Pen	Water consumption	Mean	**1.566**	**0.0364**	**1.427**	**0.0613**
		CV	**1.315**	0.0095	**1.241**	**−0.0309**
	Activity time	Mean	**1.279**	**0.0584**	**1.314**	**0.0590**
		CV	**1.324**	**−0.0379**	**1.359**	**−0.0335**
Sensor data— Environmental	Temperature	Mean	0.657	**0.0322**		
		CV	0.634	−0.0031		
	Humidity	Mean	0.861	0.0084		
		CV	0.403	−0.0100		
	Temperature humidity index	Mean	0.572	**0.0283**		
		CV	0.731	**−0.0175**		
	NH_3_ concentration	Mean	0.800	**0.0125**		
		CV	0.476	−0.0038		
	CO_2_ concentration	Mean	**1.118**	−0.0105		
		CV	0.264	**−0.0349**		
	Exhaust air rate	Mean	0.745	**−0.0978**		
		CV	**1.097**	**−0.0355**		
	Outdoor temperature	Mean	0.666	**0.0243**		
		CV	0.395	**0.0212**		
(b)						
			**All Variables**		**without Environmental** **Variables**	
			**R^2^ = 0.62**		**R^2^ = 0.65**	
			**β = 0.0526 ^1^**		**β = 0.2102 ^1^**	
**Recorded**	**Variable**	**Feature**	**VIP**	**β**	**VIP**	**β**
Direct observations— Pen	Tail posture		**3.927**	**0.7055**	**4.113**	**0.7423**
	Skin lesions		**1.062**	0.0033	0.907	0.0329
	Respiratory tract		**1.095**	−0.0201	**1.090**	−0.0384
	Gastrointestinal tract		0.288	0.0272	0.331	0.0650
	Musculoskeletal system		**1.264**	**0.0992**	**1.436**	0.1247
	Treatment index rearing		0.242	−0.0277	0.413	−0.0644
	Treatment index fattening		0.600	−0.0402	0.828	0.0126
	Weight end of rearing		**1.019**	0.0322	**1.212**	0.0626
Sensor data— Pen	Water consumption	Mean	**1.450**	0.0096	**1.033**	0.0148
		CV	0.472	0.0113	0.887	0.0108
	Activity time	Mean	**1.697**	**−0.0826**	**1.521**	−0.0376
		CV	**1.855**	**0.0690**	**1.690**	0.0444
Sensor data— Environmental	Temperature	Mean	0.881	−0.0054		
		CV	**1.095**	0.0130		
	Humidity	Mean	0.763	0.0384		
		CV	0.889	**0.0821**		
	Temperature humidity index	Mean	0.908	−0.0045		
		CV	**1.115**	0.0004		
	NH_3_ concentration	Mean	0.975	**−0.1544**		
		CV	0.870	0.0111		
	CO_2_ concentration	Mean	**1.026**	0.0279		
		CV	0.858	−0.0084		
	Exhaust air rate	Mean	**1.300**	**−0.0673**		
		CV	**1.113**	**0.0973**		
	Outdoor temperature	Mean	**1.119**	0.0014		
		CV	**1.069**	−0.0275		

^1^ VIP ≥ 1 and a │β│ ≥ β* = median (β)/interquartile range (β) are bold.

## Data Availability

The data presented in this study are available on request from the corresponding author. The data are not publicly available due to privacy concerns.
